# Comparison of the Potential of “Green” Classical and Natural Deep Eutectic Solvents in the Production of Natural Food Colorant Extracts from the Roots of *Alkanna tinctoria* (L.)

**DOI:** 10.3390/foods14040584

**Published:** 2025-02-10

**Authors:** Zvezdelina Yaneva, Neli Grozeva, Mima Todorova, Mariana Kamenova-Nacheva, Plamena Staleva, Neli Memdueva, Milena Tankova Tzanova

**Affiliations:** 1 Department of Pharmacology, Animal Physiology, Biochemistry and Chemistry, Faculty of Veterinary Medicine, Trakia University, 6000 Stara Zagora, Bulgaria; zvezdelina.yaneva@trakia-uni.bg; 2 Department of Biological Sciences, Faculty of Agriculture, Trakia University, 6000 Stara Zagora, Bulgaria; n.grozeva@trakia-uni.bg (N.G.); neli.memdueva.21@trakia-uni.bg (N.M.); 3 Department of Plant Sciences, Faculty of Agriculture, Trakia University, 6000 Stara Zagora, Bulgaria; mima.todorova@trakia-uni.bg; 4 Laboratory for Extraction of Natural Products and Synthesis of Bioactive Compounds, Research and Development and Innovation Consortium, Sofia Tech Park JSC, 111 Tsarigradsko Shose blvd., 1784 Sofia, Bulgaria; mariana.nacheva@orgchm.bas.bg (M.K.-N.); plamena.staleva@orgchm.bas.bg (P.S.); 5 Institute of Organic Chemistry with Centre of Phytochemistry, Bulgarian Academy of Sciences, Acad. G. Bonchev Str., bl. 9, 1113 Sofia, Bulgaria

**Keywords:** alkanet, antioxidants, GC-MS, HPLC-PDA-MS, natural deep eutectic solvents, natural food colorants, UV/Vis spectrophotometry

## Abstract

*Alkanna tinctoria* L. Tausch (“alkanet” or “alkanna”) is a medicinal plant. Extracts from alkanet roots have applications as natural food coloring agents. In addition, they have proven antioxidant effects. Three classical solvents (ethanol and acidified ethanol/water) and four natural eutectic solvents (NADES)—choline chloride/urea; choline chloride/citric acid; choline chloride/lactic acid; and sodium acetate/formic acid—were compared for their effectiveness as “green” solvents for the extraction of the alkanet pigment. Notably, this study is the first to apply choline chloride-based NADESs for alkanet extraction, providing a comprehensive profile of key bioactive compounds and their contributions to antioxidant activity using UV/Vis and FT-IR spectrometry, GC-MS, and HPLC-PDA-MS. Among the classical solvents, 50% acidified ethanol showed the highest extraction capacity, as indicated by its total flavonoid (708 ± 32 mgCE/L) and total phenolic (1318 ± 63 mgGAE/L) content. However, this extract exhibited the highest total alkaloid content (256 ± 15 µg/L) compared to the other classical extraction solvents. Consequently, absolute ethanol was identified as a more suitable alternative. Among the NADES, the sodium acetate/formic acid (1:2 mol/mol, NADES4) extract was the only one to show the presence of alkannins. This extract also contained high levels of phenols (355 ± 21 mg GAE/L) and tannins (163 ± 10 mg CE/L), exhibited strong antioxidant potential (DPPH: 131 µmol TE/g dw, FRAP: 7.49 mg Fe(II)/mg dw), and contained significantly lower alkaloid levels (7.0 ± 0.5 µg/L). Comparative analyses indicated that the sodium acetate/formic acid extract outperformed those prepared with ethanolic solvents and other NADES.

## 1. Introduction

*Alkanna tinctoria* L. Tausch (called also “alkanet” or “alkanna”) is a medicinal plant common in Southern Europe (including Bulgaria), Northern Africa, and Southwestern Asia [1]. It grows in dry, sunny grasslands in the lowlands. The Bulgarian populations are strongly fragmented and isolated from each other, comprising scattered solitary individuals or small groups of plants, and the species is included in the Red Book of the Republic of Bulgaria in the category “endangered” [2]. Alkanna is protected by the Biodiversity Act, and its collection from the Bulgarian population is not allowed. Some localities are within sites of the European ecological network Natura 2000 in Bulgaria [3].

The plant has blue flowers and dark red roots [4]. The roots are a proper source for the production of alkannin pigment—a mixture of naphthoquinone derivatives [5]. In addition, these extracts showed antioxidant; sun protection; anti-wrinkle; and antimicrobial effects [4,6,7]. The published results confirm a promising application in cosmetics and pharmacy. In the food industry, extracts obtained from alkanet roots have applications as natural coloring agents [8]. The structures that cause the intense red coloration are naphthoquinones—alkannin and its derivatives [6]. Due to their antioxidant [9] and antibacterial activity [10], alkanet extracts display significant preservative effects and could be promising preservative additives in the production of functional foods, e.g., meat products [10], alkannin-enriched yogurt [11], and even sweets and beverages with enhanced color and flavor [12].

*A. tinctoria* also accumulates pyrrolizidine alkaloids (PAs), of which 7-angoylretronecin, triangularin, and dihydroxy triangularin have already been identified [13]. They can be co-extracted, and the use of the extracts in the food industry can be restricted. Because of their known genotoxic and carcinogenic properties, PAs are regarded as undesirable substances in food and feed and are the subject of two EFSA opinions issued in 2007 and 2011 [14].

However, alkanet extracts are mixtures of natural biological active compounds, which work in synergy, and no scientific report on the toxicity profile of alkanet roots was found in the scientific literature [15]. Moreover, extraction can be selective when the right solvents and conditions are selected [16], and the pyrrolizidine alkaloid content of the extracts obtained can be minimized.

Green (ecological friendly) solvents are the new trend in phytochemistry. When these solvents are also selective and effective, they can lead to production cost savings [16]. Such promising solvents are the natural deep eutectic solvents (NADESs). They are a mixture of a hydrogen bond donor (HBD) and a hydrogen bond acceptor (HBA) of naturally occurring compounds that produces a low melting point solvent that is nontoxic, renewable, cheap, and easy to prepare [17]. In the extraction of polyphenols, they often showed higher extractability performance when compared to the conventional solvents since NADESs can form a strong intramolecular structure with the dissolved compounds [18,19], and the extracts obtained often had stronger antioxidant potential [16]. A big disadvantage of NADESs and a limiting factor affecting their applicability is their high viscosity, which can be adjusted via the addition of water [20,21].

Data in the world scientific literature about the use of NADESs for the extraction of alkanet colorants are scarce. Only a few deep eutectic solvents have been investigated: lactic acid and sucrose in a mol ratio of 5:1 and 30% water [22]; levulinic acid and glucose in a mol ratio of 5:1 and 20% water [23]; and sodium acetate and formic acid or lactic acid as HBD and 20% water [19]. To the best of our knowledge, no data have been published on the use of choline chloride-based NADES for alkanet extraction. No data could be found on the HPLC analysis of extracts from *A. tinctoria* obtained using NADES either.

In the search for environmentally friendly solvents for the extraction of natural colorants, and due to the limited knowledge of the effectiveness of NADESs for natural alkannin extraction, our research team set the following goal: to compare the potential of green classical and natural eutectic solvents in the production of natural food colorant extracts from the roots of *A. tinctoria*.

## 2. Material and Methods

### 2.1. Materials

#### 2.1.1. Biological Materials

The object of this research is the roots of *A. tinctoria*. The herb, due to its limited distribution of the species in Bulgaria and the strict ban on its collection from natural habitats, has been purchased from the company “Bilki Carmen” Ltd, Sofia, Bulgaria. The certificate of quality and conformity states that the herb was collected from a population of the species in Albania in the autumn of 2022 and is usable until 31 March 2026. The results of the microbiological test on the indicators *Escherihia coli*, coliforms, *Salmonella* sp., the total number of aerobic and facultatively anaerobic microorganisms, and molds indicate that the herb fully meets the microbiological criteria for quality and safety. The quantity of the presently found aflatoxins is below the threshold of acceptance. The presence of heavy metals Pb, Cd, and Hg has not been established. The moisture content has been determined to be 11.3% *w*/*w*.

#### 2.1.2. Chemicals

The chemicals, analytical chromatographic-grade reagents and solvents applied in the present study were purchased from Sigma-Aldrich (Saint Louis, MA, USA). They include the following: DPPH (2,2-diphenyl-1-(2,4,6-trinitrophenyl)hydrazyl, C_18_H_12_N_5_O_6_, CAS No.: 1898-66-4), ABTS (ABTS™ chromophore, diammonium salt, C_18_H_18_N_4_O_6_S_4_·(NH_3_)_2_, CAS No: 30931-67-0), ethanol (EtOH, C_2_H_5_OH, p.a. ≥ 99.8%), NaOH (p.a., HPLC), HNO_3_ (p.a., HPLC), HCl (ACS reagent, 37%), phosphate-buffered saline (PBS, P-3813), K_2_S_2_O_8_ (CAS No. 7727-21-1ACS reagent, ≥99.0%), Folin–Ciocalteu’s phenol reagent, Na_2_CO_3_ (powder, ≥99.5%, ACS reagent), ascorbic acid (C_6_H_8_O_6_, CAS No: 50-81-7), acetic acid (CH_3_COOH, CAS No.:64-19-7, glacial, ACS reagent, ≥99.7%), urea (NH_2_CONH_2_, CAS No.: 57-13-6, ACS reagent, 99.0–100.5%), citric acid (HOC(COOH)(CH_2_COOH)_2_, CAS No.: 77-92-9, ACS reagent, ≥99.5%), lactic acid (CH_3_CH(OH)COOH, CAS No.: 50-21-5, ACS reagent, ≥85%), formic acid (HCOOH, CAS No.: 64-18-6, ACS reagent, ≥88%), choline chloride ((CH_3_)_3_N(Cl)CH_2_CH_2_OH, CAS No.: 67-48-1 ≥99%), sodium acetate (CH_3_COONa, CAS No.: 127-09-3, ACS reagent, ≥99.0%), Trolox (C_14_H_18_O_4_, CAS No.: 53188-07-1, 97%), ferric 2,4,6-tripyridyl-s-triazine (TPTZ, C_18_H_12_N_6_, CAS No.: 3682-35-7, for spectrophotometric det. (of Fe), ≥98%), FeCl_3_ (CAS No.: 7705-08-0, reagent grade, 97%), FeSO_4_.7H_2_O (CAS No.: 7782-63-0, ACS reagent, ≥99.0%), gallic acid ((HO)_3_C_6_H_2_CO_2_H, CAS No.: 149-91-7, ACS reagent, ≥98.0%), NaNO_3_ (CAS No.: 7631-99-4, ACS reagent, ≥99.0%), AlCl_3_ (CAS No.: 7446-70-0, reagent grade, 98%), (±)-catechin hydrate (C_15_H_14_O_6_·xH_2_O, CAS No.: 7295-85-4), vanillin (4-(HO)C_6_H_3_-3-(OCH_3_)CHO, CAS No.: 121-33-5, ≥97%), KCl (CAS No.: 7447-40-7, ACS reagent, 99.0–100.5%), bromcresol green (C_21_H_14_Br_4_O_5_S, CAS No.: 76-60-8, ACS reagent, dye content 95%), atropine (C_17_H_23_NO_3_, CAS No.: 51-55-8, certified reference material, pharmaceutical secondary standard), chloroform (CHCl_3_, CAS No.: 67-66-3, for analysis EMSURE^®^ ACS, ISO, Reag. Ph Eur). All solvents used for chromatography were of LC-MS grade.

### 2.2. Sampling and Extract Preparation

#### 2.2.1. Ultrasound-Assisted Extraction Using Classical Solvents

The plant material was ground in a mechanical grinder to a final particle size of less than 400 μm. The samples were stored in the dark at a temperature of T = 16–18 °C prior to analysis.

The amount of each dried and ground sample was weighed on an analytical balance (approx. 3 g) and suspended in a solvent at a ratio of 1:10 *w*/*v*. Classical solvents based on ethanol (ethanol; 50% ethanol adjusted to pH 2 using conc. acetic acid; 20% ethanol, adjusted to pH 3 using conc. acetic acid) were selected due to their effectiveness against plant colorants and low environmental impact [16]. The extraction was carried out via ultra-sonication for 30 min at 40 °C at 80 W/m^3^. The extraction technique of ultrasonication was selected due to the quantity of the extraction of the target compounds [24].

To perform a chromatographic analysis and measure the total content of alkaloids, the prepared extracts were lyophilized at −40 °C in a Biobase freeze dryer (Biobase Bioindustry Ltd., Jinan, China).

#### 2.2.2. Natural Deep Eutectic Solvent (NADES) Extraction

**Figure 1:**
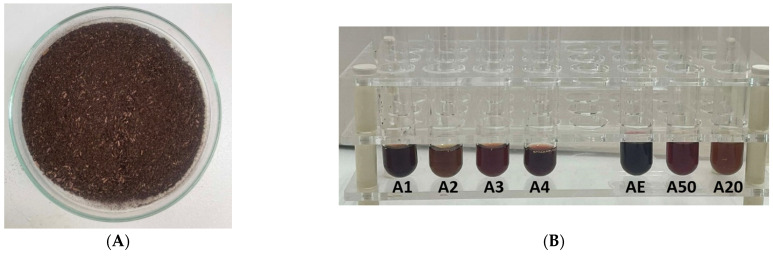
(**A**) *Alkanna tinctoria* (L.) dried roots and (**B**) ethanol and NADES alkanet extracts.

The preparation procedure of the NADESs was based on the heat-stirring method (Table 1). The NADES extracting agents were prepared by mixing a hydrogen bond acceptor (HBA) with a hydrogen bond donor (HBD) at a 1:2 molar ratio in round-bottom flasks equipped with a magnetic stirrer. For the NADES based on choline chloride and citric acid, 30% *w*/*w* of ultrapure water was added to keep its viscosity low enough at room temperature. The eutectic solvents were stirred for 20 min to 1 h within a temperature range of 50–80 °C. Once a clear, homogeneous liquid was formed without any precipitates, the NADESs were placed in glass containers that were sealed hermetically and stored at room temperature, away from light, until they were ready for further use. A predetermined amount of plant sample was weighed using an analytical balance (approx. 3 g) and suspended in a solvent at a ratio of 1:20, *w*/*w*. The extraction process was carried out by mixing the dried samples and the NADESs in a water bath for 60 min at 50 °C, followed by centrifugation for 35 min at 5300× *g* using a Heraeus Labofuge 200 centrifuge (Thermo Fisher Scientific, Waltham, MA, USA) (Figure 1).

### 2.3. Spectrophotometric Determination of the Radical Scavenging Activity

To determine the antioxidant potential of the investigated extracts, three methods with different mechanisms of action were applied: (i) DPPH radical scavenging activity [25]; (ii) an ABTS^•+^ assay [26]; and (iii) the FRAP method [27].

#### 2.3.1. 2,2-Diphenyl-1-picrylhydrazyl (DPPH) Method

Briefly, 100 μL of the extract was added to 3.9 mL of a 100 M DPPH solution in methanol. After 30 min, the absorption at 517 nm was measured using a UV/Vis spectrophotometer (Thermo Scientific Evolution 300, Thermo Ekectron Scientific Instruments LLC, Madison, WI, USA). Although the extracts are characterized by complex composition, their radical scavenging capacity was compared to Trolox, and the results were calculated through regression analysis of the linear relationship between Trolox concentration and absorption at 517 nm (with R^2^ = 0.9984 within the concentration range of 5–50 μmol/L). The results were expressed as μmol of Trolox equivalent (TE) per 1 L of extract. 

#### 2.3.2. 2,2′-Azino-bis(3-ethylbenzothiazoline-6-sulfonic Acid (ABTS) Assay

The ABTS^•+^ cation radicals were generated by reacting a 7 mM ABTS solution in distilled water with 2.4 mM K_2_S_2_O_8_ in the dark for 24 h at 20 °C, using a 1:1 *v*/*v* ratio. The ABTS solution was then diluted with absolute ethanol to achieve an absorbance of 0.700 at 734 nm, which was measured using a UV/Vis spectrophotometer (5000 DR Hach Lange, Düsseldorf, Germany). To assess the ABTS radical scavenging ability, 200 µL of the sample was added to 3.6 mL of the ABTS solution, and the absorbance was measured at 734 nm. The activity was expressed as the percentage of ABTS radical inhibition, and the radical scavenging capacity was calculated using Equation (1).

(1) $$ABTS\left(\%\right)=\left(1-\frac{I_{x}}{I_{o}}\right)*100,$$

Here, *I_x_* is the absorbance of the control, and *I_o_* is the absorbance of the sample.

#### 2.3.3. Ferric-Reducing Antioxidant Power (FRAP) Assay

The FRAP reagent was added to 0.2 mL aliquots of the extracts. The reagent had been prepared by mixing 100 mL of 300 mM sodium acetate buffer (pH = 3.6), 10 mL of 10 mM TPZT, and 10 mL of 20 mM FeCl_3_. The mixture was incubated for 30 min at 37 °C. Absorbance was measured at 593 nm, and the results were expressed as mg equivalents of FeSO_4_ per 1 mg of dry weight. A calibration curve for FeSO_4_ was created, covering concentrations from 0.1 to 1.0 mM FeSO_4_.

### 2.4. Determination of Total Phenolic Content (TPC)

The procedure for quantifying the total phenolic content (TPC), as outlined by Yaneva et al. (2024) [28], involved mixing 1 mL of plant extract with 5.0 mL of Folin–Ciocalteu’s reagent (1:10 dilution), followed by the addition of 4 mL of 7.5% Na_2_CO_3_ to the mixture, which was then left at room temperature for 60 min. A spectrophotometer (Thermo Scientific Evolution 300, Thermo Ekectron Scientific Instruments LLC, Madison, WI, USA) was used to measure the absorbance at 765 nm. A blank sample was used as a reference. A calibration curve (R^2^ = 0.9996) was generated using ethanol gallic acid solutions within the concentration range of 10 to 150 μg/mL. The TPC was expressed as milligrams of gallic acid equivalents (GAE) per liter of plant extract.

### 2.5. Determination of Total Flavonoid Content (TFC)

The procedure described by Dinev et al. (2021) [29] was followed for the quantification of total flavonoid content (TFC). In brief, 1 mL of plant extract was mixed with 0.3 mL of 5% NaNO_3_ and 4 mL of deionized water. After 5 min, 0.3 mL of 10% AlCl_3_ was added, followed by 2 mL of 1 M NaOH after 6 min. The solution was subjected to homogenization, and the absorbance was measured at 510 nm against a blank using a Thermo Scientific Evolution 300 spectrophotometer. A calibration curve (R^2^ = 0.9989) was constructed using standard catechin hydrate solutions (10–150 mg/L). The TFC was expressed as milligrams of catechin equivalent (CE) per liter of extract.

### 2.6. Determination of Total Condensed Tannin Content (TCT)

The total condensed tannin content (TCT) was measured using vanillin as the reagent and catechin as the standard, following the procedure described by Rebaya et al. (2015) [30]. In brief, 0.4 mL of the extract was mixed with 3 mL of 4% vanillin methanol solution and 1.5 mL of conc. HCl. The solution was homogenized and incubated for 15 min, after which the absorbance was measured at 500 nm against a blank using a Thermo Scientific Evolution 300 spectrophotometer (Thermo Ekectron Scientific Instruments LLC, Madison, WI, USA). Standard catechin hydrate solutions (10–150 mg/L) were used to construct the calibration curve (R^2^ = 0.9997). The TCT was expressed as milligrams of catechin equivalent (CE) per liter of extract.

### 2.7. Determination of Total Anthocyanin Content (TAntC)

The total anthocyanin content (TAntC) was determined using the pH differential method described by Lee et al. (2005) [31]. Alkanet samples were mixed with 20 mL of two buffer solutions: pH 1.0 (0.025 M potassium chloride buffer) and pH 4.5 (0.4 M sodium acetate buffer). The mixtures were incubated for 20 min at room temperature, and then centrifuged at 4 °C and 12,000 rpm for 15 min. After removing the supernatant, the absorbance was measured at wavelengths of 520 and 700 nm. The TAntC was calculated using Equations (2) and (3) as follows:

(2) $$TAntC,\frac{mg}{L}=\frac{A*.D_{F}*1000}{\epsilon *L}=\frac{A*449.2*D_{f}}{26.9}$$

(3) $$A={\left(A_{520}-A_{700}\right)}_{pH=1}-{\left(A_{520}-A_{700}\right)}_{pH=4.5}$$

where *A*_520_ and *A*_700_ are the absorbances at 520 nm and 700 nm, respectively; *M_R_*—the molecular weight of cyanidin-3-glucoside (449.2 g/mol), which is used as a reference compound; *D_F_*—the dilution factor; *ϵ*—the molar absorptivity of cyanidin-3-glucoside (26,900 L/mol.cm); and *L*—the path length of the cuvette (1 cm).

TAntC was expressed as milligrams of cyanidin-3-glucoside equivalents (CGE) per 1 L of extract.

### 2.8. Determination of Total Alkaloid Content (TAlkC)

The TAlkC was obtained via the spectrophotometric method using bromcresol green (BCG) as the reagent and atropine as the standard [32]. In brief, an aliquot amount of each lyophilized extract was dissolved in 2N HCl and filtered, and the filtrate was washed three times in 10 mL of chloroform. The pH of the non-organic layer was adjusted to 7 using 0.1N NaOH. Then, 5 mL of BCG solution (69.8 mg + 3 mL 2N NaOH + 5 mL distilled water warmed up, and then adjusted to 1000 mL with distilled water) and 5 mL of pH 4.7 phosphate buffer (2M sodium sulfate adjusted to pH 4.7 with 0.2M citric acid) were added to the neutralized solution. After homogenization, the constructed colored complex was extracted with 1, 2, 3, and 4 mL of chloroform. The chloroform extracts were collected, and the volume was adjusted to 10 mL. The absorbance at 417 nm was measured against a blank sample using a Thermo Scientific Evolution 300 spectrophotometer (Thermo Ekectron Scientific Instruments LLC, Madison, WI, USA). Standard solutions of catechin hydrate (within the concentration range of 40 to 120 mg/L) were used to construct the calibration curve (R^2^ = 0.9998). The TAlkC was expressed as micrograms of atropine equivalent (AE) per 1 L of extract.

### 2.9. ATR-FTIR Spectroscopic Analyses

The FTIR spectra were recorded in the middle infrared region 550–4000 cm^−1^ using a FTIR spectrometer: TENSOR 37 Bruker (Bruker Optik GmbH, Ettlingen, Germany). An ATR vertical attachment equipped with a ZnSe crystal was used for sampling. The results are presented as absorbance.

### 2.10. UV/Vis Spectrophotometry

All spectrophotometric analyses of the investigated extracts were conducted using a UV-Vis spectrophotometer, DR 5000 (Hach Lange, Düsseldorf, Germany), supplied with 10 mm quartz cuvette cells. All absorbance spectra were recorded in the UV/Vis regions with a 2 nm slit width, 900 nm/min scan speed, and very high smoothing.

### 2.11. Identification of Bioactive Compounds via GC-MS Analysis

To 10 mg of lyophilized extract, we added 50 µL (1.00 mg/mL) of ribitol (quantitative internal standard), and the solvent was evaporated using a rotary vacuum concentrator at 40 °C. The residue was solved in 250 µL of 20.00 mg/mL methoxyamine hydrochloride in pyridine, and the mixture was warmed for 90 min at 80 °C. Then, 250 µL of silyting reactive was added and warmed for another 30 min at the same temperature. After cooling, a 1 µL aliquot was injected at a 10:1 split mode into the gas chromatographic system (Agilent GC 7890 A with mass detector Agilent MSD 5975 C, Waldbronn, Germany). The separation was carried out on an HP-5MS column (30 m, 0.32 mm ID, film thinness 0.25 µm), and the temperature program was set as follows: start at 100 °C (2 min hold), increase to 180 °C by 15 °C/min (1 min hold), and increase to 300 °C by 5 °C/min (10 min hold). The injector and detector temperatures were 250 °C; the carrier gas was helium (1.0 mL/min); and the scanning range of the MS detector was set at *m*/*z* = 50–550.

The components were identified by comparing registered mass spectra with those of the Golm Metabolome Database (http://gmd.mpimp-golm.mpg.de/) and NIST 08 database (National Institute of Standardization and Technology, Gaithersburg, MD, USA).

### 2.12. HPLC-PDA-MS Analysis

The liquid chromatographic system used was HPLC-PDA-ESI/MS on a Shimadzu LC-2040C 3D Nexera- and Shimadzu LCMS 2020 (single quadrupole). The separation of compounds was carried out using a column Accucore C18 (Thermo Fisher Scientific Inc., Waltham, MA, USA) with the following settings: 2.6 μm, 150 mm × 2.1 mm, and thermostated at 30 °C for Method 1 and 40 °C for Method 2. The UV spectra were recorded within the wavelength range of 190 to 800 nm. The ion spray voltage was set in the negative mode at −4.50 kV, with the following settings: scan range: 100–1000 *m*/*z*; interface temperature: 350 °C; desolvation line: 250 °C; heat block: 200 °C; nebulizing gas flow: 1.5 L/min; and drying gas flow: 15 L/min.

Data analysis was performed using LabSolution software (Version 5.97 SP1, 2008–2019 Shimadzu Corporation, Kyoto, Japan). The identification of these compounds was achieved via a comparison of the retention time, UV/Vis, and mass spectral data with those of the standards, literature data, and available online databases.

The lyophilized extracts, AE, AE50, and AE20, were dissolved in methanol at a concentration of 1000 mg/L. The extracts A1, A2, A3, A4, and NADES 1 to NADES 4 were diluted in methanol at a 1:3 ratio and stirred for 15 min in an ultrasonic bath. All solutions were filtered through 0.22 μm PTFE syringe filters before analysis. Additionally, the solvents used for extraction (NADES 1 to NADES 4) were injected separately.

#### 2.12.1. Identification of the Pigment Compounds—Alkannines (Method 1, M1)

The solvents used for the analysis were (A) 0.1% formic acid in water and (B) acetonitrile. The following gradient program was performed: 60% B isocratic for 10 min, 60–90% B over 5 min, 90% B isocratic for 5 min, 90–60% B over 3 min, and re-equilibration of the column for 7 min. The flow rate was 0.3 mL/min, and the injected volume was 5 μL for the extracts AE, AE50, and AE20 and 1 μL for the extracts A1, A2, A3, A4, and NADES 1 to 4. A blank sample was injected between each analysis to ensure accuracy. Alkannines were identified at 520 nm.

#### 2.12.2. Identification of Phenolic Compounds and Flavonoids (Method 2, M2)

The solvents used for the analysis were (A) 0.1% formic acid in water and (B) acetonitrile. The following gradient program was performed: 5% B isocratic for 2 min, 5–30% B over 23 min, 30–95% B over 5 min, 95% B isocratic for 4 min, 95–5% B over 1 min, and re-equilibration of the column for 5 min. The flow rate was 0.3 mL/min, and the injected volume was 3 μL for the extracts AE, AE50, AE20, A1, A2, A3, A4, and NADES 1 to 4. A blank sample was injected between each analysis to ensure accuracy. Phenolic compounds and flavonoids were identified at 280 and 350 nm.

### 2.13. Statistical Analysis

All measurements were performed in triplicate, and the values of the experimental results are presented as an average. The standard deviations of the means (±SD) of the experimental results were calculated. The Pearson correlation test and linear regression analysis were also applied to determine the relationships between the biological active compound contents and the antioxidant activities. The statistical tests were established using XLSTAT 2023.1. 2 (1406), Lumivero (2023).

## 3. Results and Discussion

### 3.1. Contents of Biologically Active Compounds

The *A. tinctoria* L. roots are rich in naphthoquinones, which add color [33], as well as organic acids and phenolic compounds, which add flavor to food [34]. The results from all the groups of biological active compounds measured (Table 2) are presented as equivalents in 1 L of the prepared extract, so that the results can be more easily compared for all extracts obtained in this study, especially since, according to scientific data, NADES extracts can be used directly in the food industry [17].

The highest concentrations of total phenolic compounds among the extracts prepared with classical solvents are found in the extract with 50% ethanol at pH = 2 (A50)—TPC = 1318 ± 63 mgGAE/L—and among the extracts prepared with eutectic solvents with NADES 4 (A4)—TPC = 355 ± 21 mgGAE/L (Table 2). The comparison of all used solvents established that the phenolic compounds were extracted in larger quantities by the ethanol-based solvents.

Looking for the quantitative retrieval of biological active byproducts from alkanet, several research teams used different classical solvents: water [19]; methanol [1,8,19,35]; ethanol [17,19,36]; and hexane [11,35,37]. The most effective among those listed was ethanol; Das et al. (2024) compared water and ethanol for obtaining extract from alkanet roots with potential application as a yogurt preservative, and the extract prepared with 70% ethanol showed better results, with a total phenolic content of 45 mg GAE/g dm [15].

Zannou and Koca (2020) observed the extraction effectiveness of classical vs. deep eutectic solvents and obtained a TPC of 49.71 mg GAE/g dw with 70% ethanolic extract vs. a TPC of 33.12 mg GAE/dw dm with 80% methanolic extract [19]. The researchers achieved better results by using deep eutectic solvents based on sodium acetate as HBA and lactic acid or formic acid as HBD. The combination of sodium acetate and formic acid displayed greater effectiveness—TPC = 176 mgGAE/g dw. These results correspond to the data obtained in our study (Table 2), as mentioned previously.

Siamandoura et al. (2023) reported results similar to those obtained by our research team [38]. Their study displayed that the ethanol/water 70% *v*/*v* mixture was significantly more efficient, resulting in extracts with higher TPC values and antioxidant activities than choline chloride-based NADES. The probable reason for the observed behavior could be the remarkably lower surface tension and viscosity of ethanol compared to NADES, which enhanced the release of phenolic compounds due to the intensification of their diffusivity in the classical solvent [39]. In addition, the UAE process is based on the generation of high-frequency sound waves, which create cavitation bubbles that collapse, subsequently generating microjets and intense localized shear forces that break down plant cell walls, thereby facilitating the release of phenolic compounds into the solvent. This mechanical disruption could notably increase the extraction efficiency by intensifying the mass transfer of phenolic compounds from the plant matrix into the solvent [40]. Moreover, UAE typically allows for better temperature control, while excessive heating applied in the NADES extraction procedure can lead to the degradation of some phenolics [41].

From another perspective, the higher contents of anthocyanins and alkaloids obtained via the application of NADES could be due to the more polar nature of phenols compared to that of alkaloids and anthocyanins. Scientific studies have proven that ethanol and water are characterized by remarkably low efficiency in extracting less polar compounds and disrupting vegetal cell walls and membranes [42].

Regarding the parameter of TFC, among the extracts prepared with classical solvents, A50 showed the highest concentration of flavonoids (TFC = 708 ± 32 mgCE/L), and among the NADES extracts, the favorite was A1, with TFC = 279 ± 17 mgCE/L (Table 2). Obviously, according to the experimental data, the classical solvents A50 and A20 displayed greater effectiveness with regard to flavonoid extraction compared to NADES. However, the attained extraction yield of NADES1 was higher than that of AE, while the extract A2 (choline chloride/citric acid) was characterized by the poorest extraction yield (TFC = 31.0 ± 2.0 mgCE/L). Similar results were reported by Gao et al. (2024), who observed higher extraction yields of choline chloride-based extracts than those of 30% EtOH and proved that the choline chloride/citric acid extract had the lowest flavonoid content [43]. In contrast, Zannou and Koca (2020) obtained extracts using NADES based on sodium acetate with higher flavonoid content than the 70% ethanolic samples: 4.8 and 1.93 mg ECE/g dw, respectively [19].

The highest contents of condensed tannins were found in the extract prepared using absolute ethanol (AE)—TCT = 43 ± 3 mgCE/L—and NADES 4—TCT = 163 ± 10 mgCE/L (Table 2)—which was obviously the undisputed favorite among all solvents tested.

Similar results were obtained in the extraction of total anthocyanins (Table 2): the highest concentrations were achieved with NADES 1 and NADES 4—TAntC = 25 and 26 mgCGE/L, respectively—which were up to five times greater than the values determined for A50 and A20 ethanolic extracts. The data reported by Benvenutti et al. (2022) established that choline chloride and organic acid-based NADES solutions presented anthocyanin yields in Brazilian berry processing byproducts up to 50% higher than those of conventional solvents—water and ethanol aqueous solution—which was in accordance with our results [44].

The high extraction ability of NADES toward anthocyanins could be explained by the liquid crystal theory or the binding theory. The mixture of HBA and HBD forms a polymer-like matrix where the solute can dissolve into the holes of this molecular network. According to the binding theory, significant intermolecular interactions, mostly H-bonds between HBA, HBD, and target molecules, make the solute part of the supramolecular structure of NADES [44,45]. However, if water is present in the NADES, it weakens these intermolecular interactions. This is probably the reason for the lowest anthocyanin content in the A2 extract obtained with choline chloride/citric acid/H_2_O NADES. In this respect, the present experimental data are in contradiction with the conclusions of Shishov et al. (2021), which state that polar compounds such as anthocyanins are better recovered by NADES solutions compared to pure NADES, since water also forms hydrogen bonds with these target compounds [46].

The ability of NADES to extract plant metabolites is affected by its polarity, viscosity, and pH. NADES with high viscosity (such as choline chloride/urea and choline chloride/citric acid) are characterized by lower extraction ability toward phenolic compounds compared to the more viscous Na-acetate/formic acid [47,48]. According to Saha et al. (2019), lowering the viscosity leads to the intensification of cavitation phenomena, provoking the formation of stronger H-bonds between the eutectic solvent and the solute, which in turn improves the extraction capacity and yield [49]. In addition, the better extraction performance of A4 toward TPC compared to that of A1 could be attributed to its lower pH (Table 1).

The higher extractability efficiency of A4 toward TPC could also be associated with the generation of multiple hydrogen-bonding networks during its preparation. Similar results, which were reported in the study by Zannou and Koca (2020), stated that the promoted extraction of phenolics by acid-based NaDESs could be due to the conferred strong H-bond intermolecular interactions among the components, as well as the low viscosity. Moreover, phenolic compounds are classified as hydrogen bond donors due to their ability to interact with acetate anions, which helps them to dissolve more easily and contributes to their satisfactory extraction performance [19].

As expected, the alkaloid content is higher in the acidified alcohol solvents, but the capacity of the eutectic solvents is greater, with NADES 3 having the highest total alkaloid value of 372 ± 22 µgAE/L (Table 2). A1 and A4 showed the lowest alkaloid contents—TAlkC = 5.1 ± 0.4 and 7.0 ± 0.5 µgAE/L, respectively—which could be attributed to their higher pH values. Various toxic effects have been associated with alkaloids being present in food. Toxicity could vary from mild effects, such as vomiting and nausea, to more chronic effects, such as teratogenicity and sudden death [50]. The latter facts make the NADES based on Na-acetate/formic acid (1:2), and choline chloride/urea (1:2) more suitable and applicable as solvents for the preparation of *Alkanna tinctoria* (L.) extracts for the food industry.

### 3.2. UV/Vis Spectrophotometric and ATR-FTIR Analyses of Alkanna tinctoria *(L.)* Extracts

**Figure 2:**
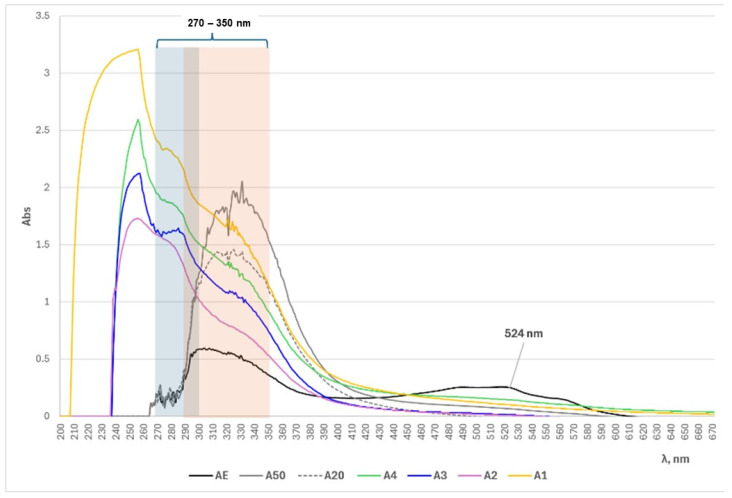
UV/Vis spectrophotometric spectra of alkanet extracts (at dilution ratio 1:23, *v*/*v*) prepared using the classical and NADES extracting agents.

According to recent scientific data, all flavonoids have aromatic chromophores, as indicated by their absorption peaks at around 250 nm in the UV region. They may undergo π, π* excitation and react to π,π* excited states. Flavonoids that contain carbonyl chromophores in their structures (e.g., quercetin) are characterized by maximum absorption in the 300 nm region. The conjugation of carbonyl chromophores with the aromatic ring, as in the molecules of acetophenones and chalcones, provokes maximum absorption in the visible region around 350 nm [51]. Considering these facts, obviously, most natural flavonoids and polyphenols are characterized by maximum absorption in the boundary UV/Vis region within the wavelength range of λ = 250–350 nm. The UV/Vis spectrophotometric analyses in the present study (Figure 2) proved the latter observations regarding the maximum absorption peaks and the conclusions of the total contents of flavonoids and polyphenols in the studied extracts, namely, the following order of increasing TFC + TPC values in the alcohol and NADES extracts: A50 > A20 > AE and A1 > A4 > A3 > A2, respectively. The shifting of the peaks of the ethanol extracts toward higher wavelengths, compared to those of the NADES extracts, could be due to the nature of the solvent and the different pH values of the solutions (Table 1).

**Figure 3:**
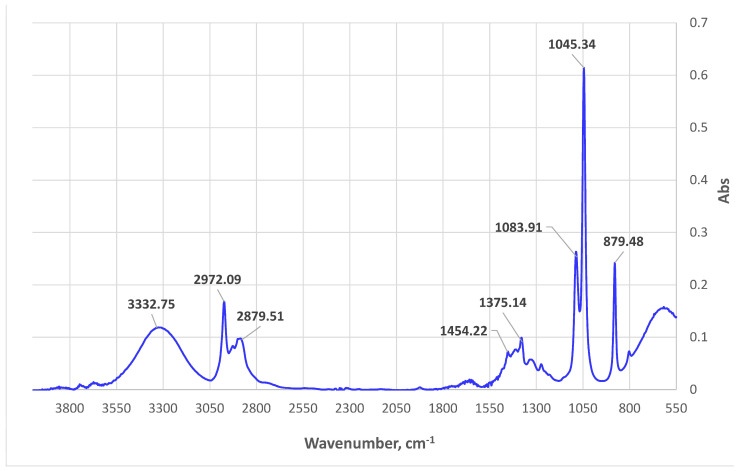
ATR-FTIR spectrum of the ethanolic alkanet extract AE.

The ATR-FTIR spectrum of AE extract is presented in Figure 3. The analysis of the assignments of the specific peaks is indicative of the presence of certain functional groups and/or chemical bonds within the molecular structures of the components of the plant extract. In this respect, the peak at 3332.75 cm^−1^ is due to the O-H stretching of alcohol. The further peak at 2972.09 cm^−1^ is indicative of C-H stretching vibrations, and the peak at 2879.51 cm^−1^ is indicative of the O-H stretching of acid components and/or symmetric vibrations of the methylene unit -CH_2_-. The shift at 1375.14 cm^−1^ is assigned to C-H/C=O stretching frequencies of extracted acids and/or C–N stretching characteristics of aromatic amines. The well distinguished peaks at 1083.91 cm^−1^ and 1045.34 cm^−1^ indicate C-H, -C-O, and -CH_3_ vibrations (rocking, deformation, and stretching), the stretching modes of C=C and C=O functional groups of organic acids, the C–O–C stretch O–H deformation of carboxyl groups, and/or N–H bond bending, which is sign of the presence of amino acids and aromatic ethers in the extract [52]. The shift at 879 cm^−1^ is due to C-H, C-O-C, C-C-O, and C-C-H deformation stretching [53,54].

### 3.3. Radical Scavenging Potential of Alkanet Extracts

**Figure 4:**
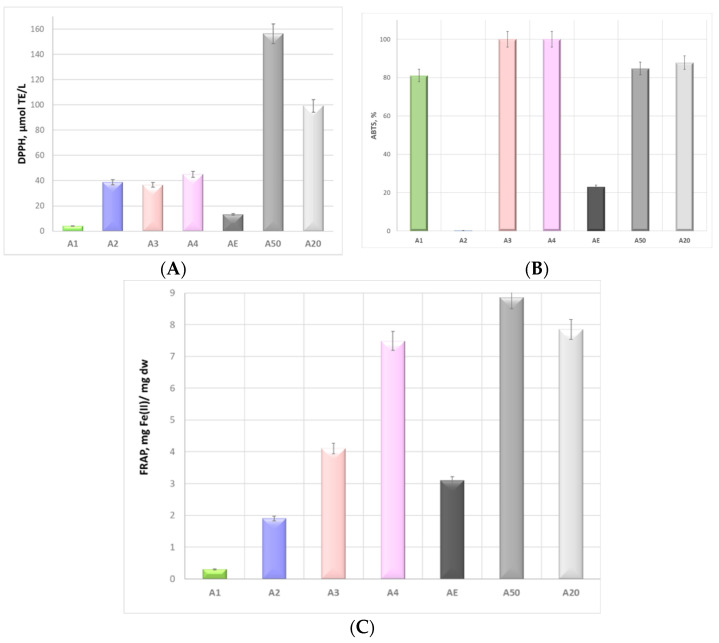
In vitro radical scavenging potential of *Alkanna tinctoria* (L.) extracts determined using (**A**) the DPPH method, (**B**) an ABTS assay, and (**C**) the FRAP method.

The antioxidant potential of *Alkanna tinctoria* (L.) extracts was measured using three methods to investigate different mechanisms of antioxidant protection: radical quenching (using the DPPH method), ferric-reducing ability (using the FRAP method), and the ability to reduce ABTS radicals (ABTS assay) (Figure 4).

The results obtained were divergent. The ethanol extract (A50) had the strongest DPPH (Figure 4A) and FRAP (Figure 4B) scavenging capacities of 156 ± 10 µmol TE/L and 8.86 mg Fe(III)/mg dw, respectively. The extracts prepared with NADES3 and NADES4 were distinguished by 100% ABTS antioxidant potentials (Figure 4C). The antioxidant potential of alkanet extract obtained with sodium acetate/formic acid achieved 131 µmolTE/L (using the DPPH method) and 7.49 mg Fe(II)/mg dw (using the FRAP method) vs. 75 µmolTE/L and 3.09 mg Fe(II)/mg dw achieved by the 70% ethanolic extracts, which the authors explained with the multiple hydrogen-bonding networks generated and the viscosity tailoring through the addition of water [19].

The comparative analyses of the antioxidant behavior of the NADES extracts revealed that the alkanet extract obtained using Na-acetate/formic acid NADES exhibited the highest DPPH and FRAP radical scavenging activities, which could be attributed to the highest contents of total phenolic compounds. The latter conclusion was consistent with the observations of other research teams, who reported that the acid-based NADESs were adequate solvents for the extraction of phenolic compounds [55,56]. As these compounds are hydrogen bond donors, they can interact with acetate anions, leading to their higher solubility and satisfactory extraction performance [57].

The positive correlation between TFC/TPC and antioxidant activity has been widely discussed in the scientific literature. Phenolic and flavonoid compounds are good reducing agents and hydrogen donors and can chelate metal ions. This makes them good antioxidant agents [8]. The correlations among the parameters measured in the present study are established in Table 3 and Table 4.

The DPPH radical scavenging capacity of the measured ethanol extracts was strongly dependent on the total phenolic content, with a correlation coefficient of 0.9937 (Table 3). The correlation between DPPH and the TFC and TAlkC of the ethanolic extracts was positive and characterized by strong correlations—R^2^ = 0.9724 and R^2^ = 0.9394., respectively The negative correlations between DPPH and TCT and TAntC were unexpected. A possible cause is the non-quantitative extraction of these compounds.

The ABTS scavenging ability of the ethanol extracts were found to be positively and strongly correlated with TPC, TFC, and TAlkC, with correlation coefficients of R^2^ = 0.9437, R^2^ = 0.7743, and R^2^ = 0.9951, respectively (Table 3), while the strongest positive dependence between ABTS and the TAntC of the NADES extracts had a correlation coefficient of R^2^ = 0.7303 (Table 4).

The correlation between the ferric-reducing antioxidant capacity (FRAP) and the total phenolic content of the extracts obtained, using both the classical and the deep eutectic solvents, was positive (Table 3 and Table 4). In the ethanolic solvents, the correlation between the FRAP and TAlkC was higher, and in the NADES, the correlation between the FRAP and TAntC was higher: R^2^ = 0.7812 (Table 3) and R^2^ = 0.6451 (Table 4), respectively.

An acidic environment could foster the stability of some phenolic compounds; thus, organic acids may assist in the regeneration of antioxidant molecules by acting as reducing agents, which could result in a more potent overall antioxidant effect [58]. Polyunsaturated fatty acids, such as eicosapentaenoic acid (EPA), docosahexaenoic acid (DHA), α-linolenic acid (ALA), and linoleic acid (LA), have a particular role in the regulation of antioxidant potential and inflammatory reactions [59]. Due to the amphiphilic nature of most polyphenolics, they exhibit both hydrophilic and lipophilic properties, which governs their cell membrane surface adsorption and/or lipid bilayer penetration abilities and subsequent interactions with hydrophobic lipid macromolecules, followed by scavenging free radicals formed during lipid peroxidation. Therefore, polyphenolic compounds can play a protective role in the cell membrane against oxidative damage, as well as modify membrane protein activity [60].

The literature presents controversial reports on the antioxidative or autooxidative effects of sugars. In this respect, some studies propose the attenuation or prevention of the detrimental effects of glucose or fructose intake through the concurrent consumption of polyphenol-rich extracts [61]. Kopjar et al. reported that sucrose and trehalose influenced antioxidant activity and had a synergistic effect on the model systems of phenolics [62].

### 3.4. GC-MS Analysis of the Alcohol Extracts

GC–MS analyses offer influence measurements of reproducibility, dynamic range, and a universal mass spectral library for small-molecular weight compounds [63].

**Figure 5:**
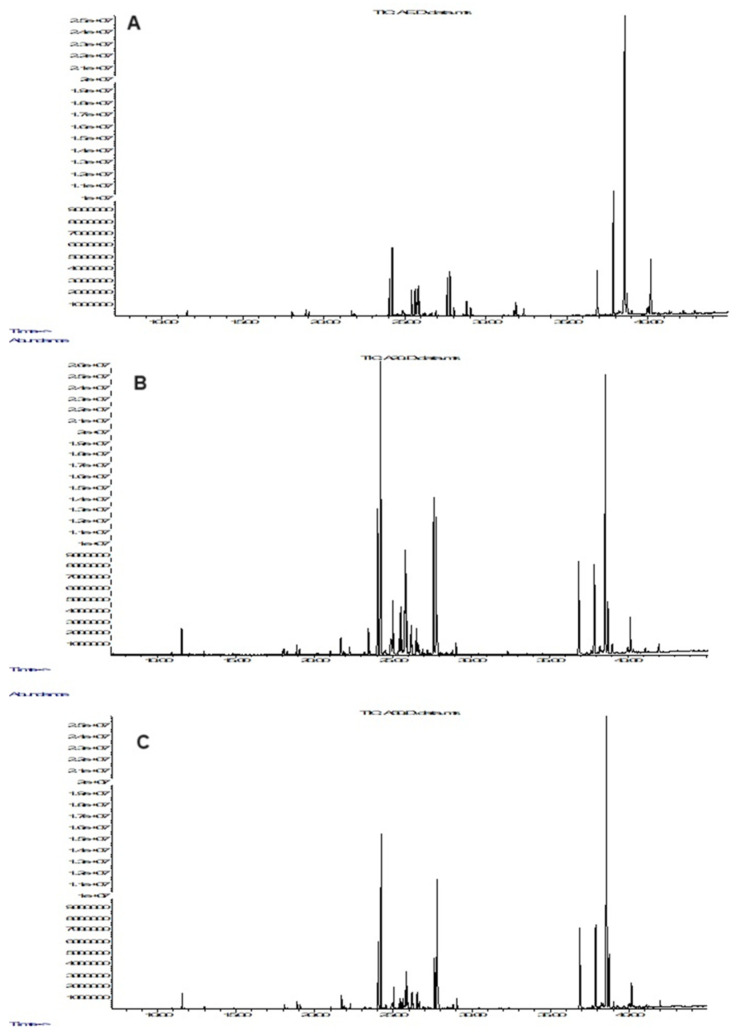
GC-chromatograms of (**A**) ethanol extract (AE; (**B**) water/ethanol (50/50) (A50) extract, and (**C**) water/ethanol (80/20) extract (A20).

Primary metabolites and byproducts were identified using GC-MS analysis in extracts obtained from the roots of *A. tinctoria* using green classical ethanolic solvents. Typical GC-chromatograms of the tested extracts are presented in Figure 5, and the results obtained are presented in Table 5. The compounds identified were grouped as alcohols; organic, phenolic, and fatty acids; and sugars. Nikolova et al. reported a similar metabolic profile of methanolic extracts from *A. tinctoria* [1].

The main alcohols present in the ethanolic extracts obtained are 4-O-methyl-myo-inositol and 6-O-methyl-myo-inositol, the highest quantities of which were detected in A20 (Table 5). Other alcohols identified were glycerol, mannitol, myo-innositol-phospates, and mono octadecanoyl glycerol, which were more efficiently extracted in the acidified ethanol/water media.

Glyceric acid was the most common organic acid, and trans-caffeic acid was the most abundant phenolic acid. Here, the tendency was the same: A20 was characterized by the highest amounts of organic and phenolic acids (Table 5). This observation is in accordance with the measured higher total phenolic contents in extracts A50 and A20 compared to the extract in absolute ethanol (Table 2).

The water/ethanolic mixture showed better extractability toward saccharides, as well. The main compound from this group was sucrose, followed by fructose and glucose (Table 5).

The fatty acids identified were palmitic and stearic acid, which were present in larger quantities in AE. Stearic acid was not detected in extracts A50 and A20 (Table 5). This is due to the fact that these organic acids are more soluble in pure ethanol than in the polar water–ethanol mixture.

### 3.5. HPLC-PDA-MS Analysis of Alkanna tinctoria *(L.)* Extracts

The experimental data obtained from the HPLC-PDA-MS analysis are summarized in Table 6.

**Figure 6:**
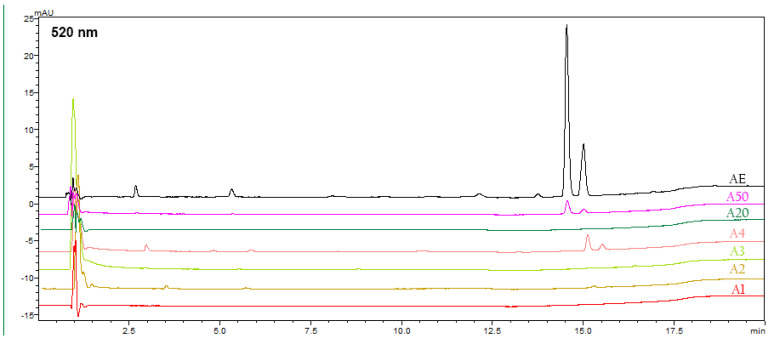
HPLC-PDA chromatograms of ethanolic and NADES extracts at 520 nm.

The results of the present study on the ethanolic and NADES extracts of *A. tinctoria* roots revealed the presence of alkannin derivatives, which are identifiable by their characteristic UV absorption maximum at 520 nm. (Table 6, Figure 6). Notably, the two characteristic peaks at 520 nm were observed exclusively in the AE, A50, and A4 extracts, while no alkannin/shikonin derivatives were detected in the other ethanolic and NADES extracts.

**Figure 7:**
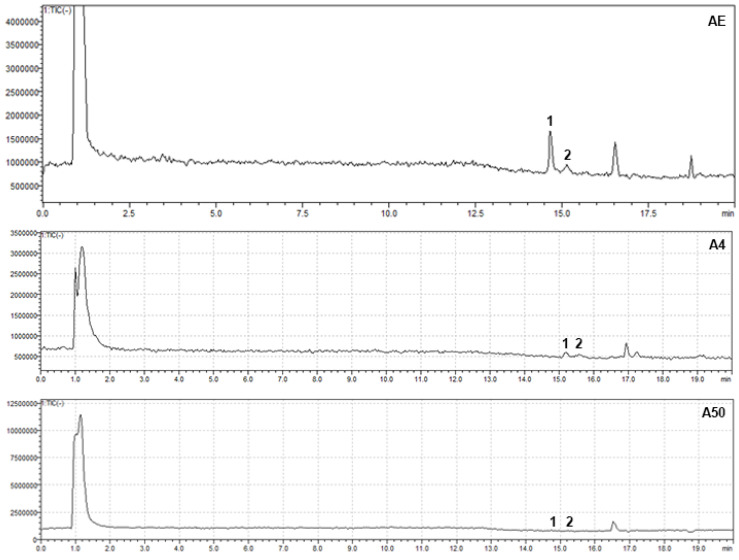
MS chromatogram of AE, A50, and A4. For the peak identification, see Table 6.

**Figure 8:**
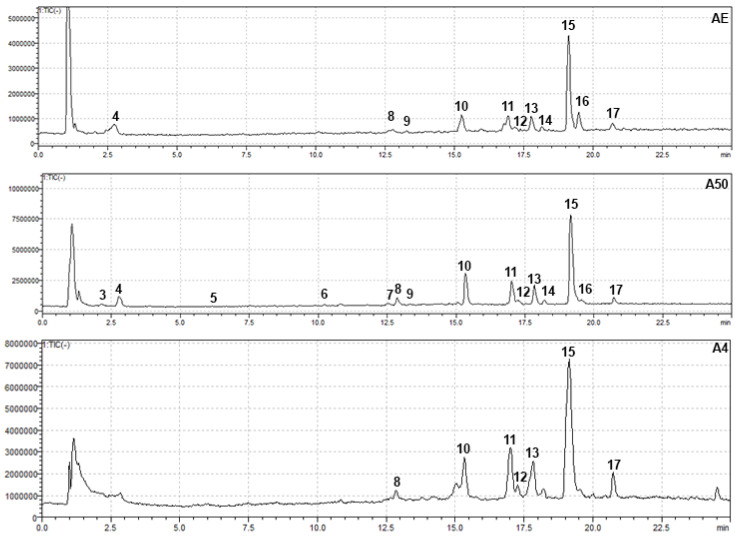
MS chromatogram of AE, A50, and A4. For the peak identification, see Table 6.

To the best of our knowledge, this is the first report of an HPLC-PDA-MS analysis of *A. tinctoria* extracts obtained using NADES (Figure 7 and Figure 8). Using ESI-MS in negative mode and referencing the literature data, the peak at Rt 14.59 min was identified as β,β-dimethylacrylalkannin, with characteristic ions [2M + Na − 2H]^−^ at *m*/*z* = 761 and [M − H]^−^ at *m*/*z* = 369. Similarly, the second peak at Rt 15.1 min was identified as isovalerylalkannin + α-methyl-n-butylalkannin, with characteristic ions [2M + Na − 2H]^−^ at *m*/*z* = 765 and [M − H]^−^ at *m*/*z* = 371. In the A4 extract, a slight shift in retention times to 15.1 and 15.5 min, respectively, was observed, which was likely due to solvent effects. These compounds have been previously reported by Assimopoulou et al. as the major hydroxynaphthoquinones detected in hexane extracts of *A. tinctoria* [37].

**Figure 9:**
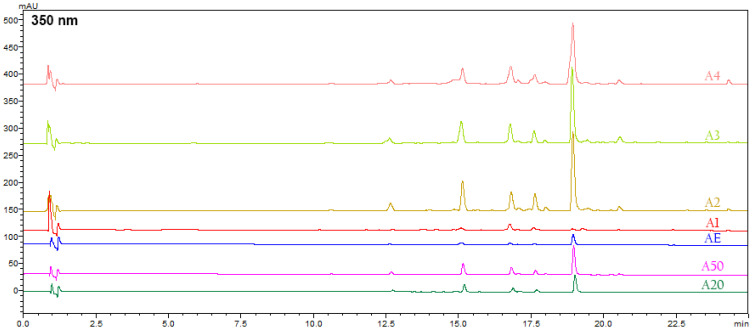
HPLC-PDA chromatograms of ethanolic and NADES extracts at 350 nm.

The second group of compounds identified in *A. tinctoria* extracts using an HPLC-PDA-MS analysis at 280 and 350 nm were phenolic compounds and flavonoids (Table 6, Figure 8 and Figure 9). Their identification was based on reference standards, the literature data, the interpretation of UV/Vis spectra, retention times, MS spectra, and open-access LC-MS libraries. A total of fifteen compounds were detected in the ethanolic and NADES extracts, with five compounds unambiguously identified as caffeic acid, syringic acid, rutin, rosmarinic acid, and salvianolic acid B using authentic standards. The compound at Rt 17.8 min was annotated as an isomer of salvianolic acid A due to the retention time difference from the standard, despite the matching *m*/*z*. Compound **8** showed a [M − H]^−^ ion at *m*/*z* = 537, corresponding to lithospermic acid [8]. Compound **10** displayed a deprotonated molecular ion at *m*/*z* = 717, which is consistent with a tetramer of caffeic acid, identified by Ganos et al. as rabdosin [8]. Compounds **14** (Rt 18.1 min), **16** (Rt 19.39 min), and **17** (Rt 20.6 min) showed the same molecular ion at *m*/*z* = 717, indicating that they are isomers of salvianolic acid or lithospermic acid [64].

## 4. Conclusions

In the search for environmentally friendly solvents for the extraction of natural colorants, and given the limited knowledge on the efficacy of NADESs for extracting alkannins from the roots of *Alkanna tinctoria*, this study compared the potential of green classical solvents (ethanol and acidified ethanol/water) and selected NADESs (choline chloride/urea; choline chloride/citric acid; choline chloride/lactic acid; and sodium acetate/formic acid). Notably, this work introduces the application of choline chloride-based NADESs for alkanet extraction for the first time, alongside HPLC-PDA-MS analysis of the extracts and a detailed assessment of the impact of different bioactive compound groups on antioxidant properties. Among the classical solvents, 50% acidified ethanol showed the highest extraction capacity based on the identified bioactive compounds, as well as the TPC and TFC values. However, this solvent also extracted alkaloids, which are undesirable for food applications, making absolute ethanol a more suitable alternative due to its lower alkaloid content. Notably, alkannins were detected only in the absolute ethanol (AE), 50% acidified ethanol (A50), and A4 extracts, while hydroxynaphthoquinone derivatives were absent in all other ethanolic and NADES extracts.

Among the NADESs, the sodium acetate/formic acid combination (1:2 mol/mol; NADES4) emerged as the most promising solvent. This extract not only contained alkannins but was also rich in phenols, flavonoids, and tannins, exhibited strong antioxidant activity, and maintained significantly lower alkaloid levels. Comparative analyses indicated that extracts prepared with NADES4 achieved better overall parameters than those prepared using ethanolic solvents.

Given that NADESs are nontoxic and enhance the stability of bioactive compounds, they offer a cost-effective and sustainable alternative for direct use in food production. However, further investigations are necessary to fully characterize sodium acetate/formic acid as an efficient and sustainable solvent for extracting alkanet-derived colorants suitable for the food industry. Studies on the cytotoxicity and antimicrobial activity of the extracts are indicated as future tasks for our research team.

## Figures and Tables

**Table 1:** Extract identification, solvent type, and preparation conditions of the solvents for the *Alkanna tinctoria* (L.) extraction.

**Extract** **ID**	**Solvent**							
	**Classical solvents**							**pH Adjusted by conc. CH_3_COOH**
AE	Ethanol							
A50	50% Ethanol at							pH = 2
A20	20% Ethanol							pH = 3
	**Natural Deep Eutectic Solvents (NADES)**							
	**NADES ID**	**HBA**	**HBD**	**H_2_O**	**Molar ratio**	**Temperature,** **T °C**	**Time,** **t, min**	**pH**
A1	NADES 1	choline chloride	urea	0%	1:2	80 °C	60	pH = 5.55
A2	NADES 2	choline chloride	citric acid	30%	1:2	80 °C	60	pH = −0.84
A3	NADES 3	choline chloride	lactic acid	0%	1:2	60 °C	20	pH = 0.66
A4	NADES 4	Na-acetate	formic acid	0%	1:2	75 °C	30	pH = 3.03

**Table 2:** Total contents of biologically active compounds in the alkanet extracts (mean ± SD, n = 3).

Sample ID	TPC	TFC	TCT	TAntC	TAlkC
	mgGAE/L	mgCE/L	mgCE/L	mgCGE/L	µgAE/L
AE	286 ± 19	203 ± 13	43 ± 3	18 ± 1	24 ± 1
A50	1318 ± 63	708 ± 32	20 ± 1	3.0 ± 0.2	256 ± 15
A20	1009 ± 41	402 ± 27	6.3 ± 0.4	4.1 ± 0.3	241 ± 13
A1	44 ± 3	279 ± 17	78 ± 6	25 ± 1	5.1 ± 0.4
A2	108 ± 7	31 ± 2	27 ± 2	4.2 ± 0.3	189 ± 11
A3	46 ± 4	38 ± 3	24 ± 1	12.2 ± 0.7	372 ± 22
A4	355 ± 21	104 ± 6	163 ± 10	26 ± 1	7.0 ± 0.5

**Table 3:** Parameter correlation matrix of alcohol/water extracts.

	DPPH	ABTS	FRAP	TPC	TFC	TCT	TAntC	TAlkC
DPPH	1	0.9006 *	0.5198	0.9937 *	0.9724 *	−0.7099	−0.9411	0.9394 *
ABTS		1	0.8394	0.9437 *	0.7743	−0.9454	−0.9945	0.9950 *
FRAP			1	0.6123	0.3061	−0.9707	−0.7781	0.7812
TPC				1	0.9400 *	−0.7844	−0.9731	0.9719 *
TFC					1	−0.5259	−0.8362	0.8334
TCT						1	0.9062 *	−0.9083
TAntC							1	−0.9999
TAlkC								1

* Values in bold are with R^2^ > 0.9000.

**Table 4:** Parameter correlation matrix of NADES extracts.

	DPPH	ABTS	FRAP	TPC	TFC	TCT	TAntC	TAlkC
DPPH	1	−0.0938	−0.1466	0.5940	−0.8894	0.1376	−0.3878	0.3593
ABTS		1	0.9866 *	0.2263	0.3058	0.4851	0.7302	−0.0782
FRAP			1	0.0739	0.2873	0.3371	0.6452	0.0470
TPC				1	−0.1647	0.8671 *	−0.9731	−0.5010
TFC					1	0.3304	0.7412	−0.7014
TCT						1	0.8109	−0.7698
TAntC							1	−0.7327
TAlkC								1

* Values in bold are with R^2^ > 0.8500.

**Table 5:** Contents of compounds identified in the ethanolic extracts via GC-MS analysis.

RI	Compound	AE	A50	A20
	Alcohols			
1287	Glycerol	0.40 ± 0.18	0.76 ± 0.22	0.86 ± 0.27
1873	6-O-Methyl-myo-inositol	1678.44 ± 21.90	1782.09 ± 25.18	2033.15 ± 32.24
1921	Mannitol	0.89 ± 0.29	1.20 ± 0.50	1.45 ± 0.70
1962	4-O-Methyl-myo-inositol	2002.26 ± 30.01	2567.16 ± 40.14	3132.26 ± 46.90
2429	myo-Inositol-1-phosphate	13.80 ± 3.22	16.23 ± 3.90	15.31 ± 4.76
2433	myo-Inositol-2-phosphate	28.83 ± 6.04	17.39 ± 4.08	14.34 ± 3.31
2779	1-Monooctadecanoylglycerol	33.70 ± 7.15	9.06 ± 3.15	12.28 ± 5.05
	Organic acids			
1341	Glyceric acid	nd	5.89 ± 1.45	4.13 ± 1.93
1490	Malic acid	4.77 ± 1.76	3.84 ± 0.93	9.52 ± 2.50
1989	Gluconic acid	0.49 ± 0.19	nd	0.79 ± 0.38
	Phenolic acids			
1628	4-Hydroxybenzoic acid	9.02 ± 2.37	16.15 ± 4.09	14.10 ± 3.78
1779	4-Hydroxy-3-methoxybenzoic acid	0.36 ± 0.14	0.97 ± 0.40	0.77 ± 0.24
1843	4-Methoxycinnamic acid	0.30 ± 0.10	0.42 ± 0.18	0.90 ± 0.32
1938	trans-p-Coumaric acid	nd	1.06 ± 0.71	0.69 ± 0.19
2100	trans-Ferulic acid	0.73 ± 0.25	0.60 ± 0.19	0.43 ± 0.15
2144	trans-Caffeic acid	24.19 ± 4.28	28.17 ± 4.07	38.92 ± 5.17
	Mono- and disaccharides			
1673	Arabinose	22.01 ± 3.12	51.28 ± 5.04	30.45 ± 4.54
1866	Fructose isomer 1	203.39 ± 21.24	284.14 ± 30.16	366.25 ± 33.12
1888	Fructose isomer 2	51.56 ± 6.71	33.18 ± 2.73	70.28 ± 7.17
1892	Manose	8.78 ± 1.72	4.11 ± 1.25	11.18 ± 2.52
1899	Glucose isomer 1	240.01 ± 15.15	200.07 ± 15.19	187.90 ± 12.41
1913	Glucose isomer 2	70.14 ± 6.93	81.28 ± 5.90	93.33 ± 7.79
1977	Glucopyranose	27.55 ± 5.86	60.31 ± 6.00	45.22 ± 3.96
2647	Sucrose isomer 1	1279.37 ± 17.04	935.41 ± 30.45	789.51 ± 20.72
2660	Sucrose isomer 2	13.59 ± 2.60	34.56 ± 2.93	20.50 ± 2.11
	Fatty acids			
2040	Palmitic Acid	111.06 ± 10.05	19.62 ± 2.56	63.02 ± 8.01
2238	Stearic acid	15.41 ± 1.88	nd	nd

The amounts of metabolites in the extracts are represented as response ratio (peak area ratios using ribitol (50 µg) as quantitative internal standard); RI—retention index; nd—not detected.

**Table 6:** Data from the HPLC-PDA-MS analysis of ethanolic and NADES extracts.

№	Compound	Rt (min) ^a^	UV–Vis (nm)	Molecular Formula	Ion Type	*m*/*z*	Extract ID							Method	Method of Identif. ^b^	Ref.
							AE	A50	A20	A1	A2	A3	A4			
1	β,β-Dimethylacrylalkannin	14.59	517, 274	C21H22O6	[M − H]^−^	369	+	+	-	-	-	-	+	M1	MS	[37]
2	Isovalerylalkannin + α-Methyl-n-butylalkannin	15.10	517, 274	C21H24O6/ C22H26O5	[M − H]^−^	371	+	+	-	-	-	-	+	M1	MS	[37]
3	Syringic acid	2.07	276	C9H10O5	[M − H]^−^	197	-	+	+	-	-	-	-	M2	MS, RS	-
4	Unknown	2.76	259			358	+	+	+	+	-	-	-	M2	MS	-
5	Caffeic acid	6.10	288, 235	C9H8O4	[M − H]^−^	179	-	+	+	-	-	-	-	M2	MS, RS	[8]
6	Unknown	10.45	278			645	-	+	+	-	-	-	-	M2	MS	-
7	Unknown	12.44	266			571	-	+	+	-	-	-	-	M2	MS	-
8	Lithospermic acid	12.80	330, 254	C27H22O12	[M − H]^−^	537	+	+	+	-	+	-	+	M2	MS	[8]
9	Rutin	13.28	355, 256	C27H30O16	[M − H]^−^	609	+	+	+	-	-	-	-	M2	MS, RS	-
10	Rabdosiin	15.27	347, 283	C36H30O16	[M − H]^−^	717	+	+	+	+	+	-	+	M2	MS	[8]
11	Rosmarinic acid	16.93	330, 248	C18H16O8	[M − H]^−^	359	+	+	+	+	+	+	+	M2	MS, RS	[8]
12	Unknown	17.15	310, 255			983	+	+	+	-	-	-	+	M2	MS	-
13	Salvianolic acid A isomer	17.80	309, 254	C26H22O10	[M − H]^−^	493	+	+	+	+	+	+	+	M2	MS	[64]
14	Lithospermic acid isomer I	18.10	353, 254, 201	C36H30O16	[M − H]^−^	717	+	+	+	-	-	-	-	M2	MS	[8]
15	Salvianolic acid B	19.10	310, 286, 253	C36H30O16	[M − H]^−^	717	+	+	+	+	+	+	+	M2	MS, RS	[8]
16	Salvianolic acid isomer I or Lithospermic acid isomer II	19.39	308, 284, 250	C36H30O16	[M − H]^−^	717	+	+	+	+	+	-	-	M2	MS	[64]
17	Salvianolic acid isomer II or Lithospermic acid isomer III	20.60	312, 283, 258	C36H30O16	[M − H]^−^	717	+	+	+	-	+	+	+	M2	MS	[64]

^a^ Retention time (Rt) was defined in UV-Vis spectrum; ^b^ MS—mass spectrometry; RS—authentic standard.

## Data Availability

The datasets presented in this article are not readily available. Requests to access the datasets should be directed to the corresponding author.
